# Modulation of the virus-receptor interaction by mutations in the V5 loop of feline immunodeficiency virus (FIV) following *in vivo *escape from neutralising antibody

**DOI:** 10.1186/1742-4690-7-38

**Published:** 2010-04-26

**Authors:** Brian J Willett, Martin Kraase, Nicola Logan, Elizabeth L McMonagle, Ayman Samman, Margaret J Hosie

**Affiliations:** 1 Retrovirus Research Laboratory, Institute of Comparative Medicine, Faculty of Veterinary Medicine, University of Glasgow, Bearsden Road, Glasgow G61 1QH, UK

## Abstract

**Background:**

In the acute phase of infection with feline immunodeficiency virus (FIV), the virus targets activated CD4+ T cells by utilising CD134 (OX40) as a primary attachment receptor and CXCR4 as a co-receptor. The nature of the virus-receptor interaction varies between isolates; strains such as GL8 and CPGammer recognise a "complex" determinant on CD134 formed by cysteine-rich domains (CRDs) 1 and 2 of the molecule while strains such as PPR and B2542 require a more "simple" determinant comprising CRD1 only for infection. These differences in receptor recognition manifest as variations in sensitivity to receptor antagonists. In this study, we ask whether the nature of the virus-receptor interaction evolves *in vivo*.

**Results:**

Following infection with a homogeneous viral population derived from a pathogenic molecular clone, a quasispecies emerged comprising variants with distinct sensitivities to neutralising antibody and displaying evidence of conversion from a "complex" to a "simple" interaction with CD134. Escape from neutralising antibody was mediated primarily by length and sequence polymorphisms in the V5 region of Env, and these alterations in V5 modulated the virus-receptor interaction as indicated by altered sensitivities to antagonism by both anti-CD134 antibody and soluble CD134.

**Conclusions:**

The FIV-receptor interaction evolves under the selective pressure of the host humoral immune response, and the V5 loop contributes to the virus-receptor interaction. Our data are consistent with a model whereby viruses with distinct biological properties are present in early versus late infection and with a shift from a "complex" to a "simple" interaction with CD134 with time post-infection.

## Background

Infection with feline immunodeficiency virus (FIV) results in a progressive immune-dysfunction characterized by a gradual decline in helper (CD4+) T lymphocytes. Clinical signs include non-resolving gingivitis-stomatitis, wasting, cachexia, neuropathological deficits, and an increased incidence of malignancy [1-9]. In the United Kingdom alone there are approximately 10 million domestic cats[10] with a seroprevalence approaching 5%[6], this equates to approximately 0.5 million FIV-infected cats. Given the similarities between the clinical outcomes of infection with FIV and the human immunodeficiency virus (HIV), FIV infection of the domestic cat is established as a valuable non-primate model for AIDS in humans, providing insights into the likely efficacy of potential vaccine strategies and facilitating the exploration of novel therapeutic interventions[11,12]. The primate lentiviruses use CD4 as a primary receptor [13-15], and the restricted expression of CD4 *in vivo *targets the virus preferentially to T-helper (T_h_) lymphocytes and cells of the monocyte/macrophage lineage. However, CD4-expression alone is insufficient to confer susceptibility to infection with HIV, which also requires co-receptors, principally the chemokine receptors CXCR4 and CCR5 [16-19](reviewed in [20]). In HIV infection, disease progression is often accompanied by a shift in the co-receptor usage of the dominant variants in the peripheral circulation, from CCR5 (and occasionally CCR3)-dependent to CXCR4-dependent viruses [21-25](reviewed in [26]). In contrast to the primate lentiviruses, all primary isolates of FIV isolated from domestic cats tested to date utilise CD134 (OX40) as a primary attachment receptor [27-29] and CXCR4 as a co-receptor [30-34] (CCR5 does not mediate infection with FIV[35]). Recent data have revealed two distinct modes of interaction between FIV and its primary receptor CD134 (reviewed in[36]). FIV strains such as GL8 and CPG require determinants in both CRD1 and CRD2 of CD134 for infection while at the other extreme, B2542 and PPR are capable of infecting via an interaction with CRD1 alone[27,37]. Preliminary analyses of the modes of CD134 interaction of diverse strains of FIV have indicated that GL8 and B2542 represent two extremes of a spectrum, with many viruses displaying an intermediate dependency on determinants within CRD2 for infection [27,36]. As yet there are no data to discern whether differences in the nature of the Env-CD134 interaction contribute to pathogenicity *in vivo*; however, there is a correlation between the nature of the Env-CD134 interaction and sensitivity to antagonism by soluble CD134L and anti-CD134[38,39].

Previously, we proposed that early infection with FIV may be dominated by viruses with a complex, high affinity interaction with CD134 (reviewed in [36,40]) and which target cells where CD134 is abundant (activated CD4+ T cells[29]) and which have a restricted cell tropism. With disease progression, variants would emerge that have a less complex, low affinity interaction with CD134 and a propensity for CD134-independent infection through a direct interaction with CXCR4. Accordingly the cell tropism of the virus may shift with disease progression leading to viral dissemination into novel cellular compartments. These variants may be controlled by the humoral immune response in early infection, but would become more abundant as the disease progressed through escape from neutralisation and exhaustion of the humoral immune response. In this study we examined the diversity of viral variants in the blood of a cat infected with a molecular clone of FIV strain GL8 (GL8_414_). As a first step towards understanding the process of viral evolution in FIV infection, we characterised the biological properties of the viral variants from this animal and demonstrated the emergence of variants with distinct receptor usages and sensitivities to neutralising antibodies. Moreover, we demonstrate for the first time that mutations in the V5 loop, a primary target for escape from neutralisation may modulate the virus-receptor interaction, suggesting that escape from neutralising antibodies may drive alterations in the virus-receptor interaction.

## Methods

### Preparation of genomic DNAs

Peripheral blood mononuclear cells (PBMC) were collected at post mortem (p.m.) from a cat (A613) infected for 322 weeks with the 414 molecular clone of FIV-GL8[41]. A613 was one of a group of 3 specific pathogen-free cats infected with the 414 clone. Following infection, the virus achieved a high proviral load in PBMC and triggered a depletion of CD4+ lymphocytes and the expansion of an activated CD8+ lymphocytes subset [41]. Retrospective analysis of PBMCs collected at post-mortem indicated that viral variants had evolved in each of the three animals[42], most notably in A611 and A613. The amplification of full-length *env *ORFs by limiting dilution PCR has been described previously[42]. In brief, 1 × 10^6 ^PBMCs were resuspended in 200 μl phosphate buffered saline (PBS), and DNA was prepared using QIAamp DNA blood mini kit (QIAgen Ltd., Crawley, U.K.) as per manufacturer's instructions. The eluted DNAs were either used immediately for limiting-dilution PCR or stored at -80°C. The amplified products were cloned into either the GL8_MYA _molecular clone or the eukaryotic expression vector VR1012 as previous[41]. A detailed analysis of mutation rates, recombination frequency and quasispecies diversity is described elsewhere[42]. In this study we focused on the quasispecies population from A613 as a preliminary analysis of a random selection of nucleic acid sequences obtained by direct sequencing of the products of the limiting-dilution PCR which indicated substantial amino acid sequence diversification. Nucleic acid sequences were ascertained by using a BigDye^® ^Terminator v1.1 cycle sequencing kit (Applied Biosystems, Warrington, United Kingdom) and analysis on an Applied Biosystems 3700 genetic analyser followed by chromatogram analysis using the Chromas v1.45 software package (Technelysium Pty. Ltd., Tewantin, Australia).

### Characterisation of env genes *in vitro*

To assess whether the *envs *encoded functional products, they were cloned into the GL8_MYA _molecular clone as Mlu-I/Nde-I fragments as previous[41] and transfected into HEK-293T[43,44] cells using SuperFect activated dendrimer (QIAgen) as per manufacturer's instructions. Virus was recovered by passage of 0.45 μm-filtered supernatant onto MYA-1 cells[45], viral growth was then monitored visually for cytopathicity and confirmed by ELISA for p24. Simultaneously, Envs were subcloned as Sal-I/Not-I fragments into pVR1012 (Vical Inc., San Diego, USA) and used to generated HIV(FIV)-luciferase pseudotypes. 5 μg of each VR1012-*env *construct and 7.5 μg of pNL4-3-Luc-E^-^R^-^[46] were co-transfected into HEK-293T cells using SuperFect. The nomenclature "HIV(FIV)" denotes an FIV Env protein on an HIV particle; reference pseudotypes bearing the GL8 and B2542 Envs have been described previously[27,28,37]. Culture supernatants were collected at 48 hours post-transfection, filtered at 0.45 μm and frozen at -80°C until required. Target cell lines were seeded at 5 × 10^4 ^cells per well of a CulturPlate™-96 assay plate (Perkin Elmer, Life and Analytical Sciences, Beaconsfield, UK) and used immediately. The cells were then infected with 50 μl of HIV(FIV) luciferase pseudotypes, cultured for 72 hours and then luciferase activity quantified by the addition of 50 μl of Steadylite HTS™ (Perkin Elmer) luciferase substrate prior to measurement by single photon counting on a MicroBeta TriLux luminometer (Perkin Elmer).

### Exchange of variable loops in chimaeric GL8 Envs

Variable loops were exchanged between *envs *in the vector GL8-414ABSN, a derivative of VR1012-GL8-414[47] which had been engineered to introduce **A**paI, **B**ssHII, **S**alI and **N**heI sites at nucleotides 1000, 1366, 1528 and 1848 respectively (facilitating the exchange of *env *fragments while reserving the integrity of the amino acid sequence). The BssHII site was introduced by PCR-directed mutagenesis using oligonucleotides 7644F 5'-ACTGAAGCGCGCTTTAGGATT-3' and 7672R 5'-CTACATCTAATCCTAAAGCGCGCTTC-3' while the NheI site was introduced with 8118F 5'-GCTATTCATGTTATGCTAGCTCTTG-3' and 8123R 5'-TTGCAAGAGCTAGCATAACATGA-3'. Fragments encompassing the V4 and V5 loops of variants B14, B19 and B28 were amplified using a high fidelity polymerase chain reaction and oligonucleotides incorporating flanking 5' BssHII and 3' NheI sites respectively (7644F and 8123R). The products were then purified, digested and cloned into BssHII/NheI digested GL8-414ABSN. The nucleic acid sequences of the chimaeric *envs *were confirmed as above and then the constructs used to prepare HIV(FIV) luciferase pseudotypes.

### Cells and viruses

MYA-1[45], CLL[27] and MCC[48] cells were cultured in RPMI 1640 medium; HEK-293T cells were maintained in Dulbecco's modification of Eagle's medium (DMEM). All media were supplemented with 10% foetal bovine serum (FBS), 2 mM glutamine, 0.11 mg/ml sodium pyruvate 100 IU/ml penicillin, 100 μg/ml streptomycin. The medium for MYA-1 cells was supplemented with conditioned medium from a murine cell line (L2.3) transfected with a human IL-2 expression construct (kind gift of M. Hattori, University of Tokyo, Japan) at a final concentration equivalent to 100 U/ml of recombinant human IL-2, and 50 μM 2-mercaptoethanol. All media and supplements were obtained from Invitrogen Life Technologies Ltd. (Paisley, UK). HEK-293T cells, and MCC and CLL cells bearing feline CD134 and its derivatives[27,49] were maintained in media supplemented with G418 (InVitrogen, Paisley, UK).

### Virus neutralising antibody (VNA) assays

Plasmas were diluted 5-fold in MYA-1 culture medium, and then 25 μl of each dilution (in triplicate) were incubated with 25 μl of HIV(FIV) luciferase pseudotype, incubated for one hour at 37°C and then added to 50 μl (5 × 10^4 ^cells) of CLL-CD134[27] per well of a CulturPlate™-96 assay plate (Perkin Elmer). The cells were then cultured for 72 hours and luciferase activity quantified by the addition of 100 μl of Steadylite HTS™ (Perkin Elmer) luciferase substrate and measurement by single photon counting on a MicroBeta luminometer (Perkin Elmer). Percent neutralisation was calculated by comparing the mean luciferase counts at each plasma dilution with the mean luciferase counts for the no plasma control.

### Inhibition of viral entry

1 × 10^5 ^MYA-1 or MCC-CD134 cells were incubated with either soluble Fc-TNC-CD134L[38] or Fc-CD134[38], or anti-CD134 (7D6)[39] in complete medium in triplicate wells of 96-well culture-treated luciferase assay plates (CulturPlate™ 96) for 30 minutes at 37°C. In assessing whether pre-incubation with Fc-CD134 enhanced CD134-independent infection, virus was pre-incubated with Fc-CD134 for 30 minutes prior to plating on the target cell line. Viral pseudotypes were then added, and the plate was returned to the 37°C incubator. Cultures were maintained for 72 hours post-infection at which point 100 μl of Steadylite HTS™ (Perkin Elmer) luciferase substrate were added and luminescence was measured by single photon counting on a MicroBeta luminometer (Perkin Elmer). Percent infection was calculated by comparing the mean (n = 3) luciferase activity of each antagonist concentration against the mean (n = 3) luciferase activity of untreated cells (100% infection).

## Results

### Characterisation of the env quasispecies from A613

**Figure 1:**
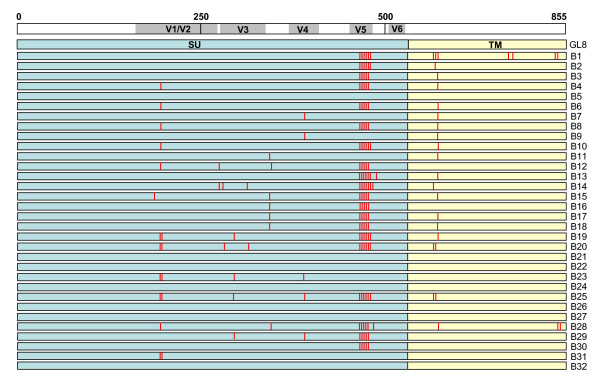
**Locations of amino acid sequence mutations in the A613-derived Env clones**. Mutations are marked by a red line in either SU (blue) or TM (yellow). Positions are shown relative to a linear representation of Env (SU and TM), amino acid numbering is indicated above.

**Figure 2:**
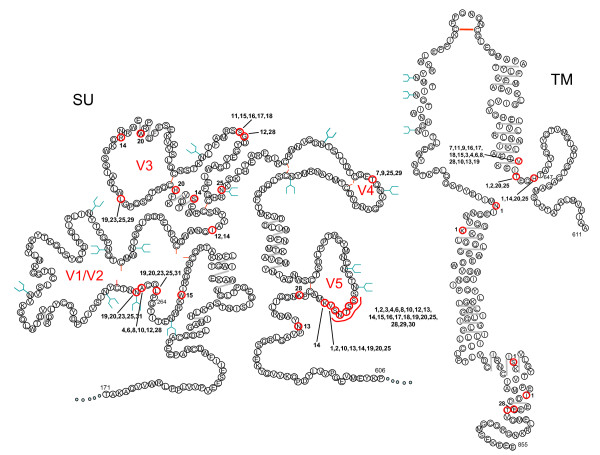
**Schematic structural model of the FIV SU and TM proteins after Pancino *et al***[67], **illustrating the locations of variations in the amino acid sequence of the A613 variants**. Mutated amino acids are circled (red) with individual clone numbers indicated. Also shown are potential sites for N-linked glycosylation (blue "Y"), di-sulphide bond formation (red line between cysteines) and predicted α-helice formation (grey line).

The early phase of infection with the GL8 molecular clone has been described previously[41]; the virus established a high proviral load rapidly, and this was maintained throughout the duration of the study[41]. Viral variants cloned from A613 harboured mutations in regions of Env thought to be associated with tropism and/or escape from neutralising antibody. It was striking that even after 322 weeks of infection, 7 species were isolated with identical amino acid sequences to the infecting strain GL8 (Fig. 1). Among the variants detected, hotspots for non-synonymous mutation were identified at the stem of the V1/V2 loop homologue (L265S, A268D, N269G and N269K) and the tip of the predicted V5 loop in SU, and in TM (H648Q, T651A, V655M) (Fig. 2). The specific targeting of the region adjacent to and including the predicted N-linked glycosylation site at 269NNT271 was of particular interest as this region had been identified previously as a determinant of the Env-CD134 interaction[49]. The range of substitutions, insertions and deletions detected in the V5 loop region was remarkable and suggestive of a strong selective pressure on this region for evolution, potentially in evasion of the host immune response. Full length *envs *encoding intact open reading frames, and which generated replication-competent viruses following sub-cloning into the pGL8 MYA molecular clone and transfection into HEK-293T cells/recovery into MYA-1 T cells, were subcloned into a eukaryotic expression vector and used to generate HIV(FIV) pseudotypes. The receptor usage and neutralisation sensitivity of the variants were then assessed *in vitro*. By comparing the distribution of amino acid sequence changes between the 32 clones, we identified 9 representative *env *clones that included the majority of the amino acid changes in Env that would allow us to distinguish the relative contributions of each variation from the parent sequence to escape from neutralisation and altered receptor usage.

### Receptor usage of Envs

**Figure 3:**
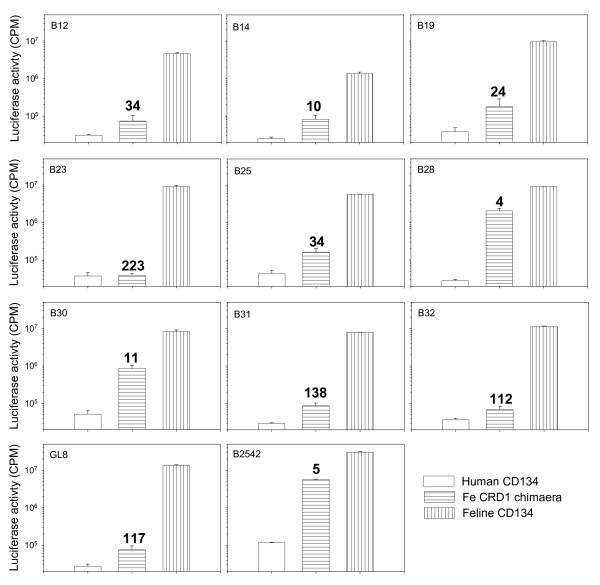
**Receptor usage of A613 variant Envs**. MCC cells stably transduced with retroviral vectors bearing human CD134, a feline CRD1 × human CD134 chimaera (feCRD1 chimaera)[27] or feline CD134 were infected with HIV(FIV) luciferase pseudotypes bearing the Envs from variants with distinct amino acid sequences, in comparison with the GL8 and B2542 Envs. The ratio of luciferase counts on feline CD134 expressing cells divided by counts on feCRD1 chimera expressing cells is shown in bold. Each value represents the mean of triplicate estimations +/- standard error and is typical of at least two independent experiments.

The GL8 strain of FIV requires determinants in both CRDs 1 and 2 of its primary receptor CD134 for infection; chimaeric receptors based on human CD134 and which express CRD1 only of feline CD134 (feCRD1 chimera) fail to support infection efficiently[27,37]. In contrast, strains such as B2542 and PPR use feCRD1 efficiently, suggesting a less stringent, or more flexible interaction with CD134. We have postulated that this "simple" interaction may evolve with disease progression, while the strains that dominate in early infection have a "complex" high affinity interaction[27,36,37]. The ability of the A613 variants to utilise native feline CD134 was compared with the feCRD1 chimera (Fig. 3). The prototypic strains with "complex" (GL8) and "simple" (B2542) interactions displayed high and low dependencies on CRD2 respectively (ratios of 117 and 5 respectively, derived by dividing the CPM on feCD134-expressing cells by the CPM on feCRD1-expressing cells). The variants isolated from the A613 quasispecies displayed a spectrum of receptor utilisations, from those similar to the GL8 parent (B23, B31 and B32, ratios of 223, 138 and 112) to those more similar to B2542 (B14, B28 and B30, ratios of 10, 4 and 11). The amino acid sequences of clones B23, 31 and 32 were identical across V5. The emergence of variants such as B28 that are able to use the feCRD1 chimera is consistent with *in vivo *evolution towards a "simple" mode of interaction.

**Figure 4:**
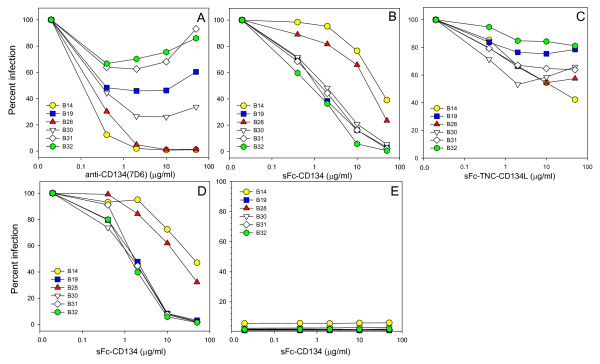
**Sensitivity of A613 Env variants with distinct receptor usages to antagonists of the virus-receptor interaction**. HIV(FIV) luciferase pseudotypes bearing the B14, B19, B28, B30, B31 and B32 Envs were examined for sensitivity to antagonism by anti-CD134 (A), sFc-CD134 (B, D, E) or sFc-TNC-CD134L (C). Infections were performed on MYA-1 cells (A-C), MCC-CD134 (D) or MCC control (E). (E) Pre-incubation with sFc-CD134 did not facilitate CD134-independent infection of MCC control cells. Each point represents the mean of triplicate estimations and is expressed as percent infection relative to no antagonist control. Each graph is typical of at least two independent experiments.

Six of the viral variants with distinct Envs were analysed further for sensitivity to antagonists of the virus-receptor interaction (Fig. 4). Variant B32 was identical to the parent strain GL8 and therefore served as the reference strain. If the nature of the virus-receptor interaction had evolved *in vivo*, it is likely that this would be reflected in altered sensitivity to antagonism by anti-CD134 antibody (7D6), soluble CD134 (sFc-CD134) or soluble CD134L (sFc-TNC-CD134L). The six variants displayed a spectrum of sensitivities to inhibition by 7D6 (Fig. 4A); B31 and B32 were relatively resistant to inhibition by 7D6, a maximal reduction of ~40% inhibition at 2 μg/ml while at high concentrations the inhibitory effect disappeared (as noted previously for GL8[39]). In stark contrast, infection with variants B28 and B14 was reduced by 70% (P = 0.014) and 90% (P = 0.003) respectively at 0.4 μg/ml and blocked completely (>99%) at all concentrations of 2.0 μg/ml or greater (P = 0.0004 at 50 μg/ml). Thus the mutations acquired by B28 and B14 *in vivo *had significantly enhanced sensitivity to antagonism by 7D6. We next asked whether the viruses differed in their sensitivities to antagonism by soluble CD134 (sFc-CD134) (Fig. 4B). Infection with B19, B30, B31 and B32 was blocked with a similar efficiency, a 50-60% reduction in infectivity being achieved at 2 μg/ml sFc-CD134 and >95% at the maximum concentration tested (50 μg/ml). These observations are in marked contrast to pseudotypes bearing the Envs of variants B14 (P = 0.0003 at 2 μg/ml) and B28 (P = 0.0002 at 2 μg/ml) which were significantly more resistant to antagonism by soluble CD134 (Fig. 4B.), indicating an alteration in the nature of the virus-receptor interaction following evolution *in vivo*. It is noteworthy that the two variants that were most sensitive to inhibition by 7D6 (B14 and B28) were most resistant to antagonism by sFc-CD134.

Previous studies demonstrated that infection with primary isolates such as GL8 and CPG41 was relatively resistant to antagonism by soluble CD134L (sFc-TNC-CD134L) while infection with strains such as B2542 and PPR was sensitive to modulation[38,39]. sFc-TNC-CD134L had a modest inhibitory effect on infection with the six viral variants however most sensitive to inhibition were B14 and B28 (Fig. 4C, significant reduction at 10 μg/ml, P = 0.004 and 0.007 respectively), consistent with the enhanced sensitivity of these two strains to anti-CD134 and reduced sensitivity to soluble CD134.

Previous studies have suggested that pre-incubation of FIV with soluble Fc-CD134 fusion proteins may facilitate infection of CXCR4-expressing (CD134-negative) cells[50,51], the studies indicating that engagement of soluble CD134 triggers a conformational change in the FIV Env that exposes the CXCR4 binding site[50,51]. Given the low sensitivity of variants B14 and B28 to antagonism by sFc-CD134 on IL2-dependent (MYA-1) T cells, we postulated that these variants may partially escape antagonism by sFc-CD134 by utilising CXCR4 for infection. To examine this hypothesis, MCC cells stably transduced with a retroviral vector bearing CD134 (Fig. 4D) or vector only (Fig. 4E) were infected with viral pseudotypes following pre-treatment with sFc-CD134. As observed with IL2-dependent T cells (Fig. 4B), sFc-CD134 blocked infection of MCC-CD134 efficiently with variants B19, B30, B31 and B32 while variants B14 and B28 proved more resistant. The sensitivity of the variants to inhibition was very similar on both MYA-1 and MCC-CD134 substrates; given that MCC-CD134 express ~100-fold more surface CD134 than MYA-1 cells[38] the data suggest that the Env-sFcCD134 interaction itself determines the degree of inhibition and that within the range examined, the level of surface CD134 expression on the target cell does not influence the outcome. When the sFc-CD134 pre-treated pseudotypes were plated onto control MCC cells (Fig. 4E), cells that are CD134-negative and CXCR4-positive[49], we observed no evidence of enhanced infection by adding increasing concentrations of sFc-CD134 (values are expressed as percent infection of control MCC cells compared with MCC-CD134). The data suggest that pre-incubation of these viruses with sFc-CD134 does not trigger exposure of the CXCR4-binding site and mediate CD134-independent infection. Thus the differential sensitivities of variants B14 and B28, and variants B19, B30, B31 and B32, to inhibition by sFc-CD134 and sFc-TNC-CD134L more likely reflects an intrinsic shift in the nature of the Env-CD134 interaction with evolution *in vivo*.

### Sensitivity to neutralising antibody

**Figure 5:**
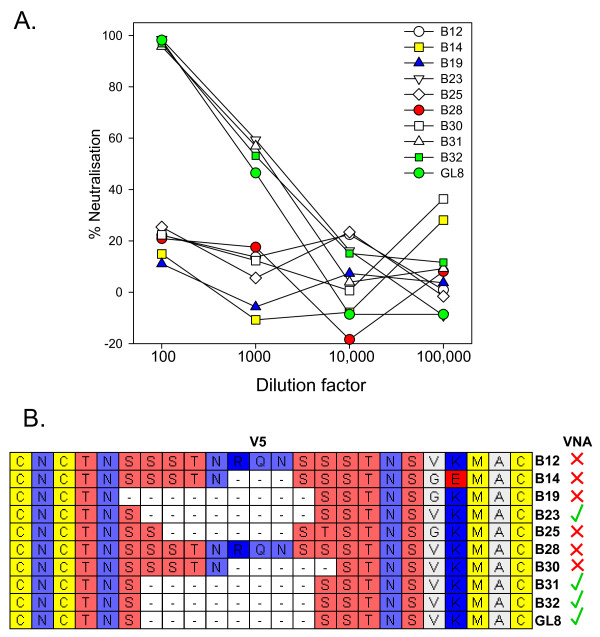
**A) Sensitivity of Env variants to neutralisation by homologous plasma from A613**. HIV(FIV) luciferase pseudotypes bearing the B12, B14, B19, B23, B25, B28, B30, B31, B32 and GL8 Envs were incubated with 1:100, 1:1000, 1:10,000 or 1:100,000 dilutions of A613 plasma and plated onto CLL-CD134 cells[27]. Luciferase activity was measured at 72 hours post-infection. Each point represents the mean of triplicate estimations and is expressed as percent neutralisation relative to the no plasma control. Each graph is typical of at least two independent experiments. **B) Neutralising activity correlates with variability in the V5 region of Env**. Amino acid residues are coloured according to chemical type: basic (dark blue), acidic (red), hydrophobic (white), hydroxyl (salmon pink), sulphur-containing (yellow) or amido (lavender blue). Presence or absence of neutralisation is summarised alongside by a green tick or red cross respectively.

Pressure from the adaptive immune response may drive the emergence of variants of HIV-1 with novel cell tropisms[52,53] while the growth of viruses in cell culture *in vitro *can select for variants with distinct neutralisation properties[54]. Given that variants had evolved *in vivo *with distinct modes of interaction with CD134, we next asked whether evasion of the humoral immune response may have provided the driving force for Env evolution. The sensitivity of nine unique viral variants to neutralisation by plasma collected post-mortem was assessed *in vitro *in comparison with the parent GL8 clone. GL8 and the closely related clones B23, 31 and 32 were neutralised effectively by the P.M. plasma (Fig. 5A). In contrast, clones B12, B14, B19, B25, B28 and B30 resisted neutralisation by the plasma. The amino acid sequences of the nine variants were compared with the GL8 clone. Although no single amino acid substitution correlated with resistance to neutralising antibody, resistance did correlate with the acquisition of mutations in V5 per se. While the V5 loops of B23, 31 and 32 were identical to GL8, those of B12, B14, B19, B25, B28 and B30 bore amino acid insertions, substitutions or deletions (Fig. 5B). The data suggest that the V5 region may be a dominant target for neutralising antibody and that antibodies targeting this region may drive immune escape in FIV infection.

### Exchange of the V5 loop confers resistance to neutralisation and modulates the virus-receptor interaction

**Figure 6:**
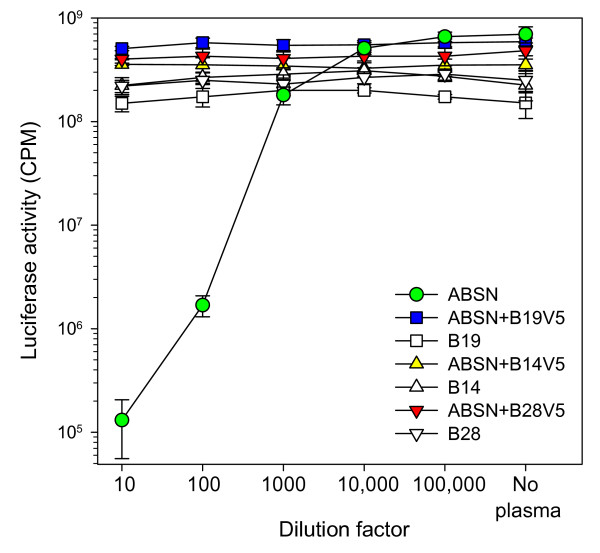
**Conferral of resistance to neutralisation by homologous plasma following V5 loop exchange**. GL8 Env incorporating non-coding mutations that introduced restriction enzyme sites facilitating V5 loop exchange (GL8 "ABSN") was compared with three chimaeric Envs in which the V5s of B14, 19 and 28 had been introduced, or the parent B14, **B**19 and B28 Envs. HIV(FIV) luciferase pseudotypes bearing each Env were incubated with 1:10, 1:100, 1:1000, 1:10,000 or 1:100,000 dilutions of A613 plasma and plated onto CLL-CD134 cells. The chimaeric Envs bearing the V5s of B14, B19 and B28 recapitulated the neutralisation-resistant phenotype of the parent Envs. Each point represents the mean (+/- SE) of three replicates and is typical of at least two independent experiments.

The data suggest that the viral variants derived *in vivo *escaped neutralising antibody by the acquisition of mutations in the V5 loop region. We therefore generated chimaeric *env*s in which a fragment encompassing the V4 and V5 loops of the parent 414 strain was exchanged with the equivalent regions (nts. 1366-1848) of the variants B14, B19 and B28; HIV(FIV) pseudotypes were prepared and the sensitivities of the chimaeric Envs to neutralising antibodies were examined. The mutated GL8 clone ABSN retained the neutralisation sensitivity of the parent clone to the A613 plasma, confirming the integrity of the amino acid sequence post-mutagenesis (Fig. 6). In contrast, substitution of the V4-V5 domain of ABSN with the corresponding domain of variants B14, B19 or B28 rendered the virus resistant to neutralisation (Fig. 6). Given that the only variation in the V4-V5 region between variants B14, B19 and B28 and the parent clone was the V5 loop itself, the data are consistent with the dominant neutralising activity of the PM plasma from A613 targeting V5.

**Figure 7:**
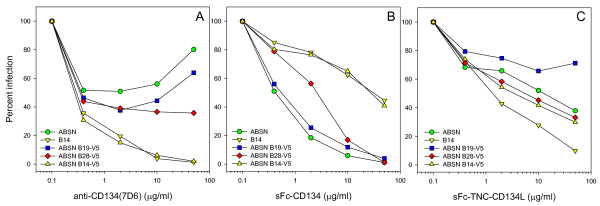
**Effect of V5 loop exchange upon sensitivity to antagonists of the virus-receptor interaction**. HIV(FIV) luciferase pseudotypes bearing the GL8 (ABSN) or B14 Envs, or ABSN+V5 loops of B14, 19 and 28 were examined for sensitivity to antagonism by anti-CD134 (A), sFc-CD134 (B) or sFc-TNC-CD134L (C). Infections were performed on MYA-1 cells. Each point represents the mean of triplicate estimations and is expressed as percent infection relative to no antagonist control. Each graph is typical of at least two independent experiments.

Following exchange of the V4-V5 region of ABSN with that of the B14, B19 and B28, we assessed the sensitivity of the chimaeric Env-bearing HIV(FIV) pseudotypes to antagonists of the virus-receptor interaction (Fig. 7). The reference GL8 Env (ABSN) conferred low sensitivity to anti-CD134 (Fig. 7A, 50.9% infection at 2 μg/ml) and high sensitivity to sCD134 (Fig. 7B, 18.4% infection at 2 μg/ml) while in contrast, the B14 Env displayed the reverse phenotype, an enhanced sensitivity to anti-CD134 (Fig. 7A, 19.5% infection at 2 μg/ml) and reduced sensitivity to sCD134 (Fig. 7B, 78.2% infection at 2 μg/ml). In comparison to the GL8(ABSN) and B14 reference Envs, the ABSN-B14-V5 chimera acquired the phenotype of the B14 Env, with high sensitivity to anti-CD134 (Fig. 7A, 6.2% infection at 10 μg/ml, significant increase P = 0.002) and low sensitivity to sCD134 (Fig. 7B, 65.1% infection at 10 μg/ml, significant reduction P = 0.002). B14 was comparatively more sensitive to inhibition by sCD134L (Fig. 7C) however exchange of V5 was insufficient to recapitulate this phenotype. The V5 loops of B19 and B28 had more marginal effects; B28 V5 was similar to that of B14 in that it enhanced sensitivity to anti-CD134 and reduced sensitivity sCD134; however, the effect was less marked than that observed with the B14-V5 (as had been observed with the B28 Env itself earlier (Fig. 4)), suggesting that determinants out with V5 may contribute to the phenotype of the B28 Env. The sensitivity of the B19 V5 chimera to antagonists of the virus-receptor interaction did not differ significantly from the GL8 ABSN parent Env. The data suggest that in the correct context, alterations in the V5 loop that confer resistance to virus neutralisation may modulate substantially the virus-receptor interaction.

## Discussion

In this study, we have demonstrated the evolution of receptor usage following infection with a homogeneous preparation of a pathogenic molecular clone of FIV. A shift in cell tropism with time post-infection has been described previously for FIV[55,56] and yet the molecular basis for such a shift has yet to be established. As all strains of FIV examined to date use CXCR4 as the co-receptor for infection, an R5 to ×4 shift analogous to that observed in HIV infection cannot be invoked. Indeed, while ×4 variants emerge in approximately 50% of HIV-infected individuals and their detection is often associated with disease progression [21-23], progression to AIDS may also occur in the absence of ×4 variants suggesting that there is not a causal link between the presence of ×4 variants and the development of AIDS. Further, the expanded cell tropism of ×4 variants may come at a cost to the virus in that ×4 variants have been observed to be targeted more readily by neutralising antibodies and may be kept in check by the humoral immune response in early infection. Given that determinants of cell tropism and virus neutralisation may be shared, pressure for escape from neutralising antibodies may drive alterations in receptor usage and cell tropism[52,53]. In this study, we present the first evidence for the evolution of the FIV-CD134 interaction *in vivo*. The animal examined in this study was infected with a homogeneous stock of a molecularly-cloned virus (GL8), a virus known to induce a decline in CD4+ lymphocytes, achieve a high viral load and to have a "complex" interaction with CD134. At post-mortem approximately six years post-infection, variants were isolated from peripheral blood of the cat that were resistant to neutralisation by homologous plasma and which had an altered interaction with CD134. It is notable that even six years post-infection we were still able to amplify *envs *that were identical to the challenge virus. The diversity of FIV is likely to result from a combination of the infidelity of reverse transcriptase, inter-variant recombination and the cytidine deaminase activities of APOBECs, with additional selective pressure from the host immune response and the level of receptor and co-receptor expression upon a target cell population. While the homogeneity of the challenge virus may have reduced the complexity of the quasispecies observed at post-mortem, we were afforded a unique opportunity to observe the shift in receptor usage *in vivo *that we had predicted *in vitro*. Why should FIV alter the way it interacts with CD134? Our studies with antagonists of the CD134 interaction suggest that the affinity of the Env for the primary receptor CD134 may alter as a consequence of escape from neutralising antibody. Surprisingly, we have uncovered mutations in the highly variable V5 loop of FIV Env that contribute significantly to the virus receptor interaction.

Sequential isolates of HIV and SIV have demonstrated the evolution of co-receptor usage *in vivo *with disease progression [24,57-61] as predicted by the frequent isolation of CXCR4 variants in symptomatic individuals[21]. Less well-studied are the alterations in the interaction between lentiviruses and their primary receptor. Intrapatient alterations in the V1V2 and V3 regions of HIV Env can modulate not only co-receptor usage but also binding to the primary receptor CD4[62]. Given that FIV uses a single co-receptor (CXCR4) for infection, the observation that the interaction between the virus and its primary receptor evolves *in vivo *may provide a mechanism whereby the virus can expand into additional cellular compartments.

Variants B14 and B28 were able to use CRD1 of feline CD134, displayed enhanced sensitivity to antagonism by anti-CD134 antibody or soluble Fc-TNC-CD134L and were more resistant to inhibition by sFc-CD134, suggesting that the evolution of the virus-receptor interaction may not simply be a modulation of binding affinity, rather it could represent a shift in the way the Env and receptor interact. The two modes of interaction may be considered as either "complex" (requiring CRD1 and CRD2) or "simple" (requiring CRD1 only)[36]. It is possible that the "complex" interaction involves trimeric Env engaging with trimeric CD134 and is of high affinity while the "minimal" interaction results from Env binding to either trimeric or monomeric CD134. Thus the latter phenotype of virus would be more sensitive to inhibition by monomeric/dimeric sFc-CD134. As receptor multimerisation may be promoted by ligand association, or by anti-CD134 antibody treatment, the viruses engaging in a complex interaction may selectively target cells exposed *in vivo *to soluble CD134L (for example helper T cells in proximity to CD134L-expressing antigen presenting cells). In contrast, viruses interacting through CRD1 alone may be suppressed *in vivo *by endogenously produced soluble CD134L.

Many of the variants isolated at post-mortem were resistant to neutralisation by homologous plasma and each resistant variant harboured mutations in the V5 loop of Env. Exchange of V5 loops between the parent virus and the late variants confirmed that neutralisation resistance was afforded by the V5 mutations. A comparison of neutralization sensitivities on three different cellular substrates (MYA-1, MCC-CD134 and CLL-CD134) gave similar findings (not shown) suggesting that the targeting of V5 was not a vagary of the assay system employed to measure VNA. The recognition of a linear peptide from variant B28 by the post-mortem plasma from A613 (not shown) confirms that the region is immunogenic, however, as we were unable to detect binding of antibodies within the A613 plasma to linear peptides derived from the neutralizable parent clone, the epitope targeted by the VNA is likely to consist of conformational epitopes encompassing V5. Previous studies have emphasised the importance of epitopes within V4 and V5 as targets for neutralising antibody and the way in which a single epitope may dominate within polyclonal sera [63-66]. Understanding the process of immune evasion may yield valuable insights into the design and optimisation of candidate vaccine immunogens that induce a protective immune response.

## Competing interests

The authors declare that they have no competing interests.

## Authors' contributions

EM, MK and NL performed the DNA manipulations, cell culture, viral pseudotype preparation and entry assays. AS assisted with the virus neutralisation assays. MH and BW designed the experiments and wrote the manuscript. All authors read and approved the final manuscript
